# Supplementary material for: Comparative Effect of Seed Coating and Biopriming of Bacillus aryabhattai Z-48 on Seedling Growth, Growth Promotion, and Suppression of Fusarium Wilt Disease of Tomato Plants
Source: Microorganisms. 2024 Apr 14;12(4):792. doi: 10.3390/microorganisms12040792 (PMC11052163; doi:10.3390/microorganisms12040792)
Supplement: Supplementary file 1 [file microorganisms-12-00792-s001.zip › microorganisms-2943779-supplementary.pdf]

Table S1: Wilt scoring of *Fusarium oxysporum*.

Score	Description
0	No symptoms
1	less than 25 % leaves wilted
2	25–50 % leaves wilted
3	51–75 % leaves wilted
4	More than 75 % leaves wilted or plants dead
